# Therapeutic effect of Internal iliac artery ligation and uterine artery ligation techniques for bleeding control in placenta accreta spectrum patients: A meta-analysis of 795 patients

**DOI:** 10.3389/fsurg.2022.983297

**Published:** 2022-09-01

**Authors:** Ayman Essa Nabhan, Yossef Hassan AbdelQadir, Yomna Ali Abdelghafar, Muataz Omar Kashbour, Nour Salem, Abdelrahman Naeim Abdelkhalek, Anas Zakarya Nourelden, Mona Muhe Eldeen Eshag, Jaffer Shah

**Affiliations:** ^1^ Faculty of Medicine, Al Andalus University, Tartus, Syria; ^2^ International Medical Research Association (IMedRA), Cairo, Egypt; ^3^ Faculty of Medicine, Alexandria University, Alexandria, Egypt; ^4^ Radiology department, National cancer institute, Misurata, Libya; ^5^ Faculty of Medicine, University of Constantine 3 Salah Boubnider, Constantine, Algeria; ^6^ Faculty of Medicine, Al-Azhar University, Cairo, Egypt; ^7^ Faculty of Medicine, University of Bahri, Khartoum, Sudan; ^8^ New York State Department of Health, Albany, NY, United States

**Keywords:** accreta, blood loss, internal iliac artery ligation, placenta accreta spectrum, uterine artery

## Abstract

Placenta accreta spectrum (PAS) can cause complications like hysterectomy or death due to massive pelvic bleeding. We aim to evaluate the efficacy of two different arterial ligation techniques in controlling postpartum haemorrhage and minimizing bleeding complications. We searched six databases. 11 studies were finally included into our review and analysis. We graded their quality using the Cochrane tool for randomized trials and the NIH tool for retrospective studies. Our analysis showed that internal iliac artery ligation has no significant effect on bleeding control (MD = −248.60 [−1045.55, 548.35] *P* = 0.54), while uterine artery ligation significantly reduced the amount of blood loss and preserved the uterus (MD = −260.75, 95% CI [−333.64, −187.86], *P* < 0.00001). Uterine artery ligation also minimized the need for blood transfusion. Bleeding was best controlled by combining both uterine artery ligation with uterine tamponade (MD = 1694.06 [1675.34, 1712.78], *P* < 0.00001). This combination also showed a significant decrease in hysterectomy compared to the uterine artery ligation technique alone. Bilateral uterine artery ligation in women with placenta accreta spectrum can effectively reduce the amount of bleeding and the risk of complications. The best bleeding control tested is a combination of both, uterine artery ligation and cervical tamponade. These techniques may offer an easy and applicable way to preserve fertility in PAS patients. Larger randomized trials are needed to define the best technique.

## 1. Introduction

Placental abnormalities are a wide group of pathologies that lead to pregnancy complications including postpartum haemorrhage (PPH) and hysterectomy. Two of the most commonly searched and encountered abnormalities are placenta accreta and placenta previa. The estimated risk of postpartum haemorrhage caused by the placenta accreta spectrum (PAS) was 41% (1). PAS is considered the main cause of postpartum hysterectomy (2). According to the latest reports, 1 of every 400 pregnancies is diagnosed with placenta accreta spectrum disorder and the incidence is increasing due to the increased number of cesarean deliveries (CD) -its main risk factor (3, 4). Other risk factors include advanced maternal age, multiparity and placenta previa (3). Clark et al. established a significant association, which implies that the risk of developing placenta accreta in patients with previous placenta previa and one previous CD is 24% and the risk increases to 67% in patients with previous placenta previa and three or more CD (5). So far, the most generally accepted management of PAS is cesarean hysterectomy (6).

Besides the maternal bleeding that requires immediate blood transfusion and increases the risk of infections and hypersensitivity reactions, the neonates may be born preterm or of low birth weight, and their 5- minutes APGAR scores may be reduced (7). ICU administration and longer hospital stay are also reported (8). Prenatal diagnosis can help to reduce both maternal morbidity and mortality (9).

In order to preserve fertility and improve maternal outcomes, several methods were developed such as arterial occlusion either by ligation or balloon catheter or embolization. Uterine sutures and administration of either uterotonic agents or methotrexate were also suggested (10–13).

Internal iliac artery ligation is a well-established procedure that was first described by Kelly in 1894 (14) and since that time, several studies investigated its effect. Multiple trials aimed to increase its efficacy by combination with balloon catheterization or tranexamic acid (15, 16). Evidence is controversial about the efficacy and use of this procedure. In this study, we aim to evaluate its efficacy by meta-analysis mainly in reducing maternal haemorrhage and blood transfusion, then compare it to other techniques demonstrated in the literature. We also aim to check the safety of the procedure by investigating its complications.

## 2. Materials and methods

We followed the guidelines of Cochrane handbook of systematic reviews (17) and the regulations of preferred reporting items of systematic reviews and meta-analysis (The PRISMA 2020 update) (18, 19) and MOOSE guidelines (20) during the conduction of this review. (A filled form of PRISMA 2020 checklist was submitted)

### 2.1. Search strategy

We used MeSH terms to form the following search strategy [(“Placenta, Retained” OR “Placenta accreta” OR “placenta percreta” OR “ placenta increta” OR “abnormally Invasive placenta” OR “Morbidly adherent placenta”) AND (ligation) AND (“iliac artery” OR “hypogastric artery” OR “uterine artery”)] to search six databases: PubMed, SCOPUS (Title and abstract search for terms), Cochrane Central, Web Of Science, VHL and Open Grey during the period of May 2020 and updated our search in September 2021, for a further check, two authors performed a manual search by screening the references of the included studies.

### 2.2. Study selection

Our inclusion criteria were: All the reports which compared artery ligation technique either internal iliac or the uterine artery to conservative therapy or any other intervention. Trials which included only PAS patients were included in our final analysis. The main outcome was blood loss reported in means and standard deviation. The accepted study designs were: Randomized control trials (RCTs) and Cohort studies. And thus, PICO criteria for our review shall be:

Population: Pregnant women with placenta accreta spectrum disorder.
Intervention: Internal iliac artery ligation or Uterine artery ligation.
Comparison: control group or any other intervention.
Outcome: Amount of blood loss and blood transfusion and any reported complication.

We excluded case reports, conference abstracts and studies that didn't report our desired outcomes. All the included studies used standard methods for limiting blood loss such as external compression and oxytocin before residing to either artery ligation or control therapy they also excluded patients with history of bleeding disorders and anticoagulants. Studies that combined other approaches with artery ligation such as tranexamic acid or balloon occlusion were excluded from our analysis. We have gone through two steps to select the eligible studies, (1) Title and abstract screening (2) full-text screening, authors were grouped into two groups and each group performed the screening and data collection separately. The first author resolved the disputes and compared the results from the two groups. The second and the last authors were primarily responsible for data analysis and writing.

### 2.3. Data extraction

We extracted the data from the included studies in two Excel sheets, in the first one, two authors extracted baseline characteristics of the eligible patients: Age, BMI, parity, gravidity, number of previous cesarean sections etc. and the other contained outcomes measurement, we divided the main outcomes into (a) Primary outcomes: Blood loss (ml) and haemoglobin change(g/dl) and (b) secondary outcomes: Blood products transfusion(units), duration of surgery(min), duration of hospital stay(days) and complications such as bladder injuries, hysterectomy, isaventensive care unit admission and coagulopathy. And after finishing the task every two authors revised the other two authors' work, A. Nabhan and Y. AbdelQadir revised the entire work.

### 2.4. Risk of bias assessment

We used the Cochrane tool to assess the risk of bias in randomized trials, as described in chapter 8.5. Depending on the following items: Random sequence generation, allocation concealment, blinding of participants and personnel, blinding of outcome assessment, incomplete outcome data, selective reporting and other bias (Missing protocol or funding issues would be considered as a source of risk), each item was graded as high risk, low risk or unclear risk of bias.

The quality of the included cohort studies (prospective and retrospective) was assessed by a quality assessment tool of the National Heart, Lung, and Blood Institute (NHLBI) (21). We used the tool for observational cohort studies included in our final screening. This tool is composed of 14 questions to assess the risk of bias and confounders. These questions were answered by “yes”, “no”, “cannot determine”, “not applicable”, or “not reported” then each study was given a score to guide the overall rating of the quality as “good”, “fair”, or “poor” quality.

### 2.5. Data analysis

We used the Review Manager Software version 5.4 to perform the meta-analysis; the continuous outcomes were measured as mean difference (MD) and standard deviation (SD), and the dichotomous outcomes as risk ratios (RR) with 95% confidence interval. In case of heterogeneity (Chi-square *P* value < 0.1), a random effect model was adopted, otherwise, a fixed-effect model was employed, and we used “take one out” method to resolve the heterogeneity, in general; the results were considered significant if the P-value was less than 0.05.

## 3. Results

### 3.1. Literature search

**Figure 1:**
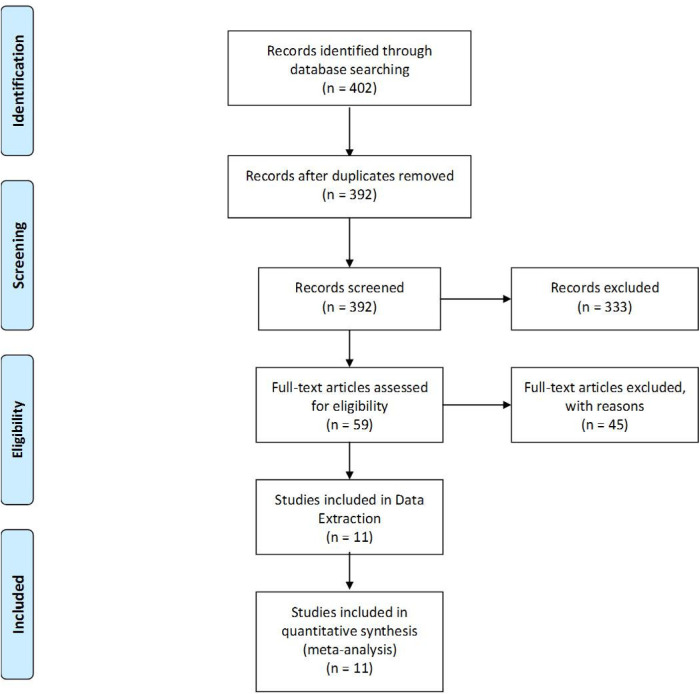
PRISMA flow chart.

The literature search retrieved 402 citations. After title and abstract screening, 59 Articles were retrieved for further evaluation (full-text screening). Four randomized trials and ten cohort studies were included. Finally, 11 studies with 759 patients were included in data extraction. (Details about the screening process is demonstrated in PRISMA flowchart Figure 1).

### 3.2. Characteristics of included studies

Baseline characteristics of the included studies including diagnosis and history of pregnancy are shown in (Table 1). Summary of the included studies and their results are shown in (Table 2).

**Table 1:** Baseline characteristics of the included studies.

Study ID	Total	Study groups	Number of patients	Age (years)	BMI (kg\m^2)	Parity	Gravidity	Number of previous cesarean sections	Gestational age (weeks)	Other Pathological findings	Pre. HgB level (g\dL)	History of placenta previa
				mean ± SD	mean	mean ± SD/ n (%)	mean ± SD	mean ± SD / *n* (%)	mean ± SD	*n* (%)	mean ± SD	*n* (%)
Iwata 2010	23	CH with IIAL	15	35 ± 3.7		1.7 ± 0.79		00:00	34.5 ± 2.1	Accreta: 4 (26.7%)		
								1:10 (67%)		Increta: 7 (46.7%)		
								2:5 (33%)		Percreta: 4 (26.7%)		
								>/=3:0				
		CH without IIAL	8	34 ± 0.6		2.5 ± 0.93		00:00	34.5 ± 2.6	Accreta: 0 (0%)		
								1:3 (38%)		Increta: 5 (62.5%)		
								2:5 (63%)		Percreta: 3 (37.5%)		
								>/=3:0				
Shamkov 2019	54	IIAL	15	31.47 ± 4.897	29.02 ± 4.75	2 ± 163	3.67 ± 2.45	1.67 ± 0.81	35.6 ± 1.5		10.92 ± 1.042	
		Temporary occlusion of CIA	18	32.72 ± 5.367	26.66 ± 4.81	1.67 ± 0.804	4 ± 2.413	1.67 ± 0.81	34.8 ± 1.6		10.79 ± 1.045	
		Combined compression	21	32.81 ± 5.046	26.37 ± 3.4	2 ± 1.59	3 ± 0.79	1.67 ± 0.81	34.3 ± 1		11.35 ± 1.198	
		hemostasis										
Hussein 2018	50	IIAL	25	33.7 ± 3.8		3.4 ± 1.1	4.5 ± 1.05	3.2 ± 1.2	36 ± 0.9		10.9 ± 0.8	
		CH only.	25	31.4 ± 4.6		3.5 ± 1.06	4.7 ± 1.2	3.24 ± 1.2	36.1 ± 0.7		10.6 ± 0.6	
Ono 2018	57	IIAL	15	34.9 ± 3.4		2 ± 1.1	2.5 ± 1.1		34.1 ± 2.5	Accreta: 4 (26.7%)		
										Increta: 8 (53.3%)		
										Percreta: 3 (20%)		
		CIA balloon occlusion;	29	33.7 ± 3.8		1.8 ± 1	2.6 ± 1.6		34.8 ± 1.5	Accreta: 17 (58.62%)		
										Increta: 7 (24.14%)		
										Percreta: 5(17.24%)		
		No occlusion	13	34 ± 3.9		1.5 ± 0.7	2.7 ± 1.1		35 ± 2.2	Accreta: 1 (7.7%)		
										Increta: 6 (46.15%)		
										Percreta: 6 (46.15%)		
Kostu 2016	45	HAL + CH	26	31.5 ± 3.1		2.5 ± 0.5	3.7 ± 0.4	2.3 ± 0.5	35.1 ± 0.8	Accreta/increta: 12 (46.1%)	11.1 ± 0.6	
										Percreta: 14 (53.9%)		
		CH only	19	33.4 ± 2.6		2 ± 0.9	3.6 ± 1.1	1.8 ± 0.7	35.8 ± 0.9	Accreta/increta: 8 (42.1%)	11.8 ± 1.2	
										Percreta: 11 (57.9%)		
Lin 2019	78	Uterine artery ligation	29	28.7 ± 7.7				1:12 (41.3%)	35.9 ± 4.2			
								2:12 (41.3%)				
								3:5 (17.2%)				
		No ligation	49	29.3 ± 6.9				1:25 (51%)	36.2 ± 4.9			
								2:19 (38.7%)				
								3:5 (10.2%)				
Elgelany 2019 .A	102	CS with cervical	48	31.8 ± 4.2		1–2:26 (54.2%)					11.4 ± 0.7	15 (31.2%)
		inversion and BUAL				3–4:22 (45.8%)							
						≥ 5:0							
		CH only	38	32.9 ± 4.1		1–2:2 (5.3%)					11.3 ± 0.3	13 (34.2%)
						3–4:22 (57.9%)						
						≥ 5:14 (36.8%)						
		Leaving placenta in place	16	31.7 ± 4.5		1:8 (50%)					11.4 ± 0.4	6 (37.5%)
						2:8 (50%)						
						03:00						
						04:00						
Martimucci 2018	37	HAL	11	34 ± 6	Prepregnancy BMI: 22.7 ± 4.9	2 ± 1.51	5 ± 2	1:2 (18%)			11.1 ± 1	9 (82%)
					BMI at delivery: 26.9 ± 5.6			2:2 (18%)				
								>3:7 (64%)				
		No HAL	26	36 ± 5	Prepregnancy BMI: 28.5 ± 6.6	2.67 ± 2.22	6 ± 3	1:7 (27%)			11.4 ± 1	20 (77%)
					BMI at delivery: 31.7 ± 6.5			2:10 (35%)				
								>3:9 (38%)				
Elgelany 2019 .B	125	Balloon tamponade and BUAL	40	26.75 ± 6.01	26.8 ± 3.5	2.5 ± 1.6		1:12 (60%)	38.1 ± 2.05	Placenta previa:	11.52 ± 0.44	Minor: 6 (15%)
								2:7 (35%)		Minor = 6 (15%)		Major: 43 (85%)
								≥3:1 (5%)		Major = 43 (85%)		
										Placental site:		
										Anterior = 36 (90%)		
										posterior = 4 (10%)		
		Only balloon tamponade	42	25.15 ± 5.13	27.3 ± 4.2	2.4 ± 1.3		1:11 (55%)	37.55 ± 2.18	Placenta previa:	11.75 ± 0.52	Minor: 11 (26.2%)
								2:8 (40%)		Minor = 11 (26.2%)		Major: 31 (73.8%)
								≥ 3:1 (5%)		Major = 31 (73.8%)		
										Placental site:		
										Anterior = 36 (85.7%)		
										posterior = 6 (14.3%)		
		Uterine artery ligation and cervical tamponade	43	25.75 ± 6.93	26.5 ± 2.5	2.7 ± 1.2		1:11 (55%)	37.8 ± 1.82	Placenta previa:	11.59 ± 0.55	Minor: 8 (18.6%)
								2:8 (40%)		Minor = 8 (18.6%)		Major: 35 (81.4%)
								≥ 3:1(10%)		Major = 35 (81.4%)		
										Placental site:		
										Anterior = 38 (88.4%)		
										posterior = 5 (11.6%)		
Barinov 2017	92	Combination of BUAL, supraplacental pleated sutures, and either excision of the tightly attached portion of placenta accreta or metroplasty	47	31.6 ± 5.5 for all participants				41(44.3%) for all participants		62(67.8%) for all participant		
		All previous surgical techniques + balloon tamponade using an intrauterine Zhukovsky catheter	20									
		All previous surgical techniques + intravaginal Zhukovsky balloon catheter	25									
Sucu 2020	96	CH with IIAL	50	32.03 ± 4.96	27.53 ± 2.68	3.36 ± 1.63	5.04 ± 1.95	2.72 ± 1.01	35.54 ± 1.2		11.43 ± 1.35	
		CH without IIAL	46	31.17 ± 4.5	27.14 ± 1.95	2.8 ± 1.15	4.22 ± 1.53	2.5 ± 0.84	35.87 ± 0.91		11.51 ± 1.11	

Data are represented as mean ± SD or *n* (%). IIAL, Internal Iliac Artery Ligation; CH, Cesarean Hysterectomy; HAL, Hypogastric Artery Ligation; BUAL, bilateral uterine artery ligation.

**Table 2:** Summary of the included studies.

Study ID	Study design	Sample size and groups.	Type and number of placenta abnormality	Intervention protocol				Report in a brief the main outcomes of the study	Hysterectomy	Success to save the uterus	Report in a brief the occurrence of complications: *n* (%)
				Targeted artery	Ligation OR occlusion	Unilateral OR bilateral	Associated interventions				
Iwata2010	Retrospective cohort	23 patients: (Ligation group: 15 patients, Control group: 8 patients)	Intervention group: Placenta Accreta = 4 (26.7%), Placenta Increta = 7 (46.7%), Placenta Percreta = 4 (26.7%). Control group: Placenta Accreta = 0 (0%), Placenta Increta = 5 (62.5%), Placenta Percreta = 3 (37.5%).	Internal Iliac artery	Ligation	Unilateral		Blood Loss (ml): ligation group = 3,721 ± 1,932, control group = 4,991 ± 2,980	All patients underwent cesarean hysterectomy		
Shmakov2019	Randomized control trial	54 patients: (IIAL ligation group: 15 patients, Temporary occlusion of the CIA: 18 patients, Combined compression hemostasis: 21 patients)	Adherent placenta = 54 (100%).	Internal Iliac artery	Ligation	Unilateral		Blood loss (ml): ligation group = 2440 ± 1215, Temporary occlusion of the CIA = 2186 ± 1353, Combined compression hemostasis = 1295 ± 520.3. Hemoglobin change (g/dl): ligation group = −1.66 ± 1.42, Temporary occlusion of the CIA = −1.68 ± 1.56, Combined compression hemostasis = −0.81 ± 1.94.	Ligation group = 1 (6.7%), CIABO group = 3(16.6%), Combined compression hemostasis group = 1 (4.8%).	Ligation group = 14 (93.3%), CIABO group = 15 (83.4%), Combined compression hemostasis group = 20 (95.2%)	Bladder injury: ligation group = 2 (13.3%), CIABO group = 0 (0%), Combined compression hemostasis group = 1 (4.8%). Bleeding in the postoperative period: (The ligation group: 0 (0%), The CIABO group: 3 (16.7%) The Combined compression hemostasis group = 1 (4.8%). Coagulopathy: ligation group = 1 (6.7%), CIABO group = 0 (0%), Combined compression hemostasis group = 0 (0%).
Hussein2018	Randomized control trial	50 patients: (Ligation group: 25 patients, Control group: 25 patients)	Abnormally invasive placenta = 50 (100%).	Internal Iliac artery	Ligation	Bilateral		Blood loss (ml): Ligation group = 1,632 ± 804, Control group = 1,698 ± 1,251. Hemoglobin change: Ligation group = 12 h:−0.77 ± 1.13/ 24 h = −0.82 ± 1.09/ 48 h = −0.96 ± 1.03. Control group = 12 h = −0.36 ± 0.68/ 24 h = −0.51 ± 0.67/ 48 h = 0.6 ± 0.65.	All patients underwent cesarean hysterectomy	None of the cases	Bladder injury: Ligation group = 8 (32%), Control group = 7 (28%).
Ono2018	Retrospective cohort	57 patients: (Ligation group: 15 patients, CIABO: 29 patients, no occlusion group: 13 patients)	Intervention group: Placenta Accreta = 4 (26.7%), Placenta Increta = 8 (53.3%), Placenta Percreta = 3 (20%). Control 1 group: Placenta Accreta = 17 (58.62%), Placenta Increta = 7 (24.14%), Placenta Percreta = 5 (17.24%). Control 2 group: Placenta Accreta = 1 (7.7%), Placenta Increta = 6 (46.15%), Placenta Percreta = 6 (46.15%).	Internal Iliac artery	Ligation	Unilateral		Blood loss (ml): Ligation group = 4,175 ± 1,921.3, CIABO = 2027.1 ± 1,637.6, no occlusion group = 3,786.7 ± 2,936.1.	All patients underwent cesarean hysterectomy	None of the cases	
Kostu2016	Retrospective cohort	45 patients: (Ligation group: 26 patients, control group: 19 patients)	Intervention group: Placenta Accreta/ Increta = 12 (46.1%), Placenta Percreta = 14 (53.9%). Control group: Placenta Accreta/ Increta = 8 (42.1%), Placenta Percreta = 11 (57.9%).	Internal Iliac artery	Ligation	Unilateral		Blood Loss (ml): Ligation group = 2204 ± 445, control group = 3,183 ± 429.	All patients underwent cesarean hysterectomy	None of the cases	Bladder injury: Ligation group = 10 (38%), Control group = 8 (42%).
Lin2019	Retrospective cohort	78 patients: (Ligation group: 29 patients, Control group: 49 patients)	Placenta Accreta = 78 (100%).	Uterine artery	Ligation	Bilateral		Blood loss (ml): Ligation group = 734.2 ± 317.5, Control group = 1,101.6 ± 442.7. Hemoglobin change (g/dl): Ligation group = −0.26 ± 0.19, Control group = −0.54 ± 0.24.	Ligation group = 0 (0%), Control group = 4 (8.2%).	Ligation group = 29 (100%), Control group = 4 (91,8%).	
Elgelany2019A	Cohort	102 patients: (Ligation group: 48 patients, Hysterectomy group: 38 patients, Preserving the placenta group: 16 patients)	Placenta Accreta = 102 (100%).	Uterine artery	Ligation	Bilateral	Cervical tamponade	Blood loss (ml): Ligation group = 2580 ± 1030, Hysterectomy group = 2840 ± 1120, Preserving the placenta group = 2120 ± 870. Hemoglobin change (g/dl): Ligation group = −1.69 ± 0.029, Hysterectomy group = −1.8 ± 0.011, Preserving the placenta group = −0.7 ± 0.011.	Ligation group = 0 (0%), Hysterectomy group = 38 (100%), Preserving the placenta group = 2 (12.5%).	Ligation group = 48 (100%), Hysterectomy group = 0 (0%), Preserving the placenta group = 14 (87,5%).	Bladder injury: Ligation group = 4 (8.3%), Hysterectomy group = 15 (39.5%), Preserving the placenta group = 1 (6.25%). Postpartum hemorrhage: Ligation group = 6 (12.5%), Hysterectomy group = 0 (0%), Preserving the placenta group = 2 (12.5%). Coagulopathy: Ligation group = 2 (4.2%), Hysterectomy group = 4 (10.5%), Preserving the placenta group = 0 (0%).
Martimucci2018	Retrospective cohort	37 patients: (Ligation group: 11 patients, Control group: 26 patients)	Placenta Percreta = 37 (100%)	Internal Iliac artery	Ligation	Bilateral		Blood loss (ml): ligation group = 1,266.67 ± 1,017.89, Control group: 816.67 ± 392.54. Hemoglobin change (g/dl): Ligation group = −0.2 ± 2, Control group = −0.8 ± 1.5.	All patients underwent cesarean hysterectomy	None of the cases	
Elgelany2019B	Retrospective cohort	125 patients: (balloon Tamponade and uterine artery ligation: 40 patients, balloon Tamponade only: 42 patients, uterine artery ligation and cervical tamponade: 43 patients)	Intervention group: Placenta accreta = 40 (100%), control 1 group: Placenta accreta = 42 (100%), control 2 group: Placenta accreta = 43 (100%).	Uterine artery	Ligation	Bilateral	Balloon Tamponade	Blood loss (ml): Balloon Tamponade and uterine artery ligation = 4580 ± 48.2, BalloonTamponade only = 4,812 ± 111.3, Uterine artery ligation and cervical tamponade = 2869.5 ± 38.38.	Balloon Tamponade and uterine artery ligation = 11 (27.5%), Balloon Tamponade only = (30.9%), Uterine artery ligation and cervical tamponade = 4(9.3%).	Balloon Tamponade and uterine artery ligation = 31 (72.5%), Balloon Tamponade only = 29 (69,1%), Uterine artery ligation and cervical tamponade = 39(90,7%).	Bladder injury: Balloon Tamponade and uterine artery ligation = 8 (20%), Balloon Tamponade only = 10 (23.8%), Uterine artery ligation and cervical tamponade = 4 (9.3%). Coagulopathy: Balloon Tamponade and uterine artery ligation = 8 (20%), Balloon Tamponade only = 9 (21.4%), Uterine artery ligation and cervical tamponade = 4 (9.3%).
Barinov2017	Cohort	92 patients: (Ligation group: 47 patients, intrauterine Zhukovsky balloon catheter group: 20 patients, Intrauterine and intravaginalZhukovsky balloon catheters: 25 patients)	Placenta Accreta = 92 (100%)	Uterine artery	Ligation	Bilateral	Barinov external supraplacental pleated sutures and either excision of the tightly attached portion of placenta Accreta or metroplasty	Blood loss (ml): Ligation group = 1681.81 ± 659.1, Intrauterine Zhukovsky balloon catheter group = 1,378.79 ± 295.45, Intrauterine and intravaginalZhukovsky balloon catheters = 1,318.18 ± 416.66	Ligation group = 26 (55.3%), Intrauterine Zhukovsky balloon catheter group = 2 (10%), Intrauterine and intravaginalZhukovsky balloon catheters = 0(0%).	Ligation group = 21 (44.7%), Intrauterine Zhukovsky balloon catheter group = 18 (90%), Intrauterine and intravaginalZhukovsky balloon catheters = 25(100%).	
Sucu 2020	Retrospective cohort	96 patients: (Ligation group: 50 patients, control group: 65 patients)	Placenta percreta = 96 (100%)	Internal iliac artery	Ligation	Bilateral		Blood loss (ml): Ligation group = 993 ± 493.43, control group = 1,019.57 ± 549.29	All patients underwent cesarean hysterectomy	None of the cases	Bladder injury: Ligation group = 9 (18%), Control group = 7 (15.2%). Pelvic hematoma leading to re-laparotomy: Ligation group = 1 (2%), Control group = 2 (4.34%)

IIAL, Internal Iliac Artery Ligation; CIABO, Common Iliac Artery Balloon Occlusion; CIA, Common Iliac Artery.

A summary of the quality assessment for the included randomized trials (22, 23) is shown in Supplementary appendix 1. Six cohort studies (24–28) were fair in quality according to NIH quality assessment tool for Observational Cohort and Cross-Sectional Studies. The other four cohort studies (16, 29–31) and were of poor quality. For more details and answers to all assessment questions in each study, (See Supplementary appendix 1).

#### 3.2.1. Internal iliac artery ligation vs. control

##### 3.2.1.1. Blood loss

###### 3.2.1.1.1. Blood loss (ml)

**Figure 2:**
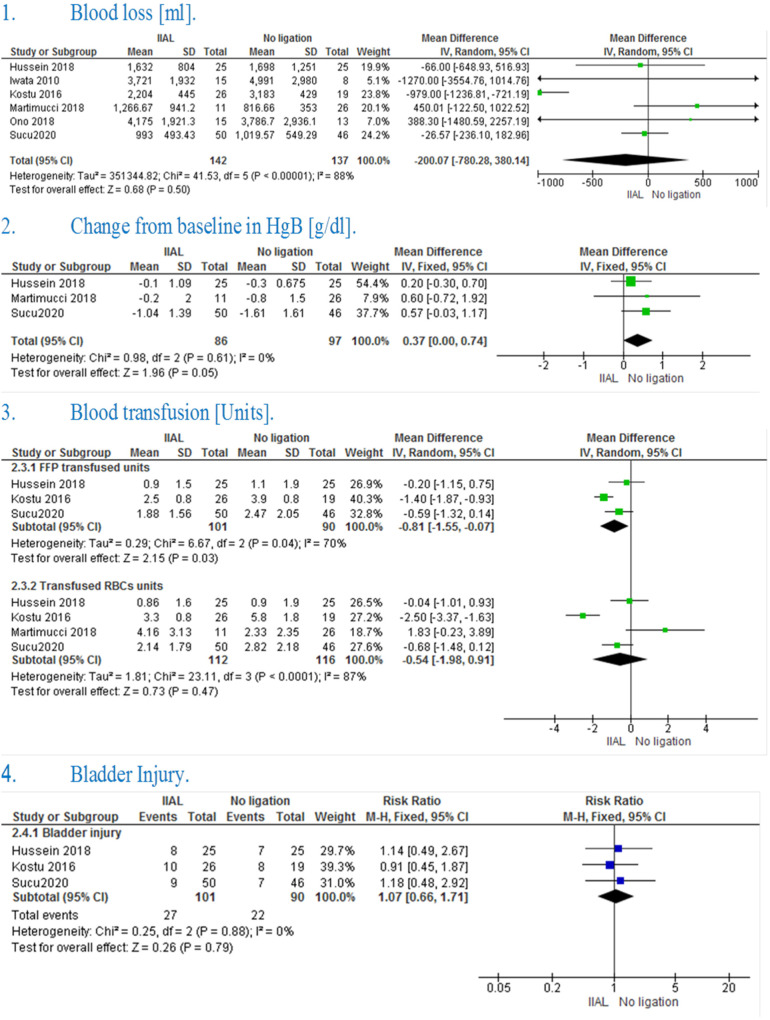
Shows the result of IIAL vs. control group.

The pooled effect estimate showed no significant difference between IIAL and no ligation (MD = −200.07 [−780.28, 380.14], *P* = 0.50). Pooled results were heterogeneous (*P**** ***< 0.00001, *I*² = 88%) and the detected heterogeneity was best resolved after excluding Kostu et al. (*P* = 0.43, *I*² = 0%) and the effect estimate remained non-significant (MD = 15.09 [−169.81, 199.99], *P* = 0.87). [Figure 2(1)]

###### 3.2.1.1.2. Change in HgB from baseline (g/dl)

The pooled studies showed a significant change in hemoglobin level from the baseline in IIAL in comparison with No ligation (MD = 0.37 [0.00, 0.74], *P* = 0.05). Pooled studies were homogenous (*P* = 0.61, *I*² = 0%) [Figure 2(2)]

##### 3.2.1.2. Blood products transfusion

###### 3.2.1.2.1. Fresh frozen plasma transfusion (units)

The pooled mean difference showed that IIAL significantly reduced the need for FFP transfusion in comparison with No ligation (MD = −0.81 [−1.55, −0.07], *P* = 0.03) The pooled studies were heterogeneous (*P* = 0.43, *I*² = 70%) and the detected heterogeneity was best resolved after excluding Kostu et al. (*P* = 0.52, *I*² = 0%) but the effect estimate became non-significant (MD = −0.44 [−1.02, 0.14], *P* = 0.13) [Figure 2(3)].

**Figure 3:**
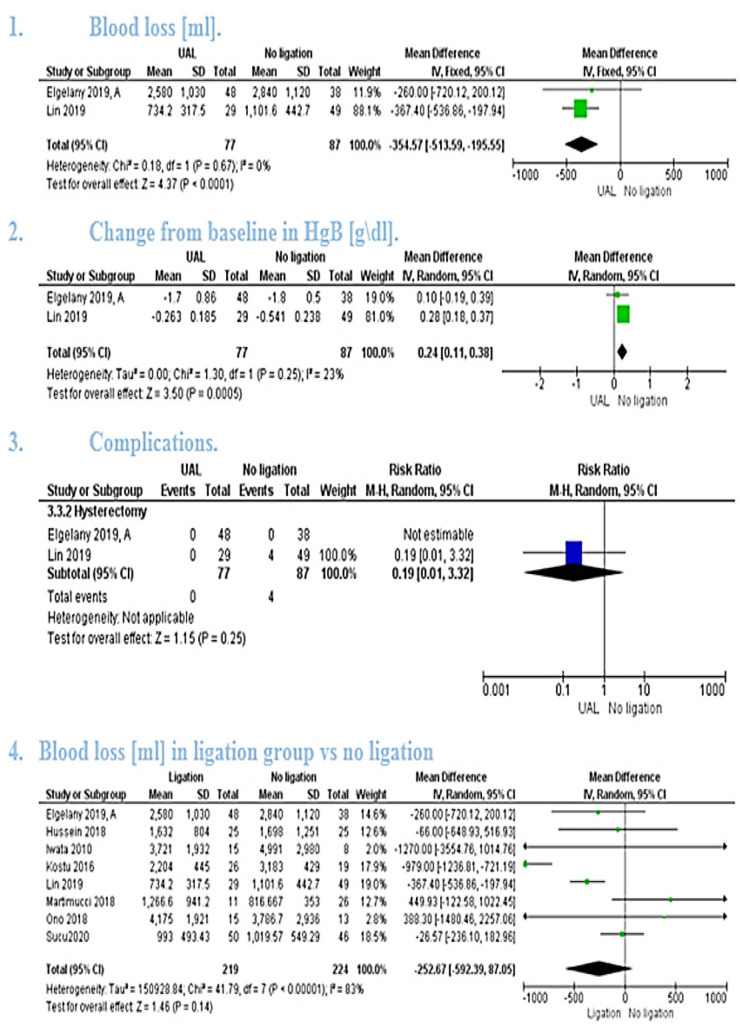
Shows the result of UAL vs. control group.

###### 3.2.1.2.2. Packed RBCs transfusion (units)

The pooled mean difference showed no statistically significant difference between the two groups (MD = −0.54 [−1.98, 0.91], *P* = 0.47).The pooled studies were heterogeneous (*P* < 0.0001, *I*² = 87%) and the detected heterogeneity couldn't be resolved [Figure 2(3)]

##### 3.2.1.3. Complications

###### 3.2.1.3.1. Bladder injury

The pooled risk ratio showed no statistically significant difference in bladder injury (RR = 1.07 [0.66, 1.71], *P**** ***= 0.79). Pooled results were homogenous (*P**** ***= 0.88, *I*² = 0%) [Figure 2(4)]

**Figure 4:**
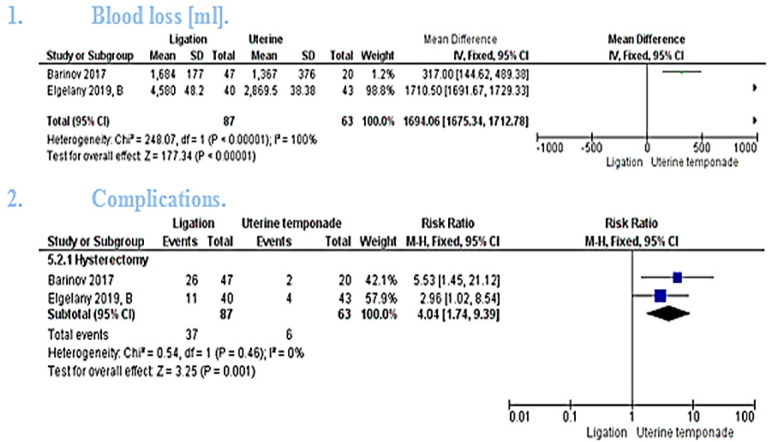
Shows the result of ligation vs. Combined uterine tamponade and ligation.

#### 3.2.2. Uterine artery ligation vs. no ligation

##### 3.2.2.1. Blood loss

###### 3.2.2.1.1. Blood loss (ml)

The pooled mean difference showed that uterine artery ligation significantly lowered the amount of blood loss (MD = −354.57, 95% CI [−513.59, −195.55], *P* < 0.0001). [Figure 3(1)] the pooled studies were homogeneous (*P* = 0.67, *I*² = 0%).

###### 3.2.2.1.2. Change in HgB from baseline (g/dl)

The pooled mean difference showed that uterine artery ligation significantly lowered the change in hemoglobin (MD = 0.24, 95% CI [0.11, 0.38], *P* = 0.0005). [Figure 3(2)] The pooled studies were homogeneous (*P* = 0.25, *I*² = 23%).

##### 3.2.2.2. Complications

###### 3.2.2.2.1. Hysterectomy

The pooled risk ratio showed no significant difference between the two groups (RR = 0.19, 95% CI [0.01, 3.32], *P**** ***= 0.25). Heterogeneity test was not applicable [Figure 3(3)].

#### 3.2.3. Artery ligation vs. control

The pooled studies showed no statistically significant difference between Artery ligation and ligation groups (MD = −252.67 [−592.39, 87.05], *P* = 0.14).The pooled studies were heterogeneous (*P* = 0 < 0000.1, *I*² = 83%) [Figure 3(4)] and the detected heterogeneity couldn't be resolved by removing a single study.

#### 3.2.4. Ligation vs. combined uterine tamponade and ligation

##### 3.2.4.1. Blood loss (ml)

The pooled mean difference showed that combined temponade and ligation cause significant reduction in the amount of blood loss (MD = 1694.06 [1675.34, 1712.78], *P* < 0.00001). The pooled studies were heterogeneous (*P* < 0.00001, *I*² = 100%) and the detected heterogeneity couldn't be resolved because only 2 studies were included in the analysis [Figure 4(1)].

##### 3.2.4.2. Hysterectomy

The pooled studies showed statistically significant higher risk ratio of hysterectomy in ligation group (RR = 4.04 [1.74, 9.39], *P**** ***= 0.001). The pooled studies were homogeneous (*P* = 0.46, *I*² = 0%) [Figure 4(2)].

## 4. Discussion

Our results showed that Internal iliac artery ligation has no significant advantage over the control group in neither blood loss (ml), haemoglobin change from the baseline nor blood elements transfusion. This corresponds with the results from the individual included studies. On the other hand, Uterine artery ligation in adherent placenta patients planned to preserve the uterus showed a significant decrease in blood loss and change in haemoglobin from the baseline. While the best blood controlling result was obtained by combining both uterine artery ligation and uterine tamponade.

Artery ligation is used in a wide variety of operations to minimize bleeding; internal iliac artery ligation has been primarily used in women undergoing hysterectomy whilst uterine artery ligation has been used in women to help save fertility. For better results, surgeons should always ligate the internal iliac artery distal to its posterior division. In complete placenta previa cases, the placental site receives a major amount of blood from the descending cervical and vaginal arteries. Even after ligation of the uterine artery, these arteries still perfuse the lower segment which causes the haemorrhage to continue. In this case, IIAL is favoured because it stops the blood flow in the uterine, cervical and vaginal vessels (32).

In order to improve the efficacy of arterial ligation, several methods were proposed. Abbas et al. investigated the addition of Tranexamic acid to bilateral uterine artery ligation (BUAL) in placenta previa patients and it greatly reduced the amount of blood loss, but the risk of thromboembolism hinders its use as prophylaxis for PPH (15). Another technique was introduced by El Gelany et al., in which he used the cervix as a natural tamponade through inverting the cervix into the uterine cavity and suturing its lips to the lower uterine segments (26). The combination of this technique with BUAL proved to be more effective than BUAL only. In another study by Elgelany et al., he advised the use of the previous technique only in patients with focal placenta accreta wishing to preserve fertility. He also offered to leave the placenta in place for patients with diffuse placenta accreta hoping to preserve fertility. Further elective laparotomy to remove the placenta is necessary, and patients may later require embolization of the uterine artery or delayed hysterectomy (16). The results of our study –result of artery ligation techniques- support the recently published article by İçen et al. which discussed the experience of a tertiary hospital in turkey which revealed that IIAL use successfully preserved fertility in 65.9% of patients of women included. Of the 70 included placenta accreta patients, the uterus could be preserved in 47 patients (33). A promising approach was suggested by Barinov et al. to preserve fertility, which reduced blood transfusion rate by 2.4 folds. He used a combination of BUAL and external supra placental pleated sutures with either excision of a small area of placenta accreta or full metroplasty and simultaneous use of intrauterine and vaginal balloon Zhukovsky catheters. This induced greater occlusion of the collateral circulation (29). Another novel technique is the use of the LigaSure instruments, which uses high frequency and low voltage electric current to occlude blood vessels, showed a significant reduction in blood loss (34).

BAKRI balloon catheter is a very simple, easily applicable and safe technique that helps reduce PPH (35). However, results published by El Gelany et al. show that it offers a nearly similar effect to the artery ligation (16). On the other hand, our results showed that a combination of uterine tamponade and ligation is more effective than ligation only (*P* < 0.00001).

Arterial balloon occlusion is another proposed technique to decrease PPH. Its main complications are lower limb ischemia and radiation exposure effect on the fetus. It's commonly done in the ILA, but results showed no significant decrease in blood loss (36). Ono et al. suggested that common iliac artery balloon occlusion (CIABO) decreases blood loss more significantly than IIA occlusion because it blocks the collateral circulation that arises from the external iliac artery during ischemia (31). He also found that CIABO decreases blood loss even more than ILAL. Wei et al. tried to improve its effect by occluding the abdominal aorta, but results showed no significant difference from internal iliac artery occlusion (37).

The choice of a wide range of diseases for testing the intervention was based on the available data. Many of the included articles didn't specify the level of placental adhesion and future research should target the effect of the intervention on each of the causes of postpartum haemorrhage. The methods of blood loss estimation differed between the included studies and some retrospective studies didn't specify exactly the method for estimation. In order to investigate the effect of such differences on our results, we performed a subgroup analysis based on the study design and the results were similar to the primary results reported in the manuscript which showed that such differences were unlikely to affect the final results and interpretation of our analysis (Supplementary Appendix 2).

Limitations of our review include 1- the limited number of randomized trials and low sample size in some included studies. 2- Patient's settings and baseline characteristics including the type of PAS differed among studies and the unknown history of bleeding disorders in some of the retrospective studies that may have contributed to the detected heterogeneity. 3- Inclusion of retrospective cohort studies alongside RCTs. 4- No protocol registration was done for this review but the steps for each stage are provided in details in the methods section under supervision of experienced and published authors.

In conclusion, uterine artery ligation can significantly reduce the amount of blood loss and hence, preserve the fertility of women with the adherent placenta. Combined IIAL and tamponade is an effective way to minimize bleeding in patients undergoing hysterectomy and might serve as an option to preserve fertility. Larger multi-centre randomized trials are needed to improve this combination and generalize the fertility saving methods.

## Data Availability

The raw data supporting the conclusions of this article will be made available by the authors, without undue reservation.

## References

[1] Mehrabadi A Hutcheon JA Liu S Bartholomew S Kramer MS Liston RM Contribution of placenta accreta to the incidence of postpartum hemorrhage and severe postpartum hemorrhage. Obstet Gynecol. (2015) 125:814–21. 10.1097/AOG.0000000000000722 25751202

[2] Usta IM Khalifeh T Nassar AH. Peripartum hysterectomy: 1999 to 2006. Obstet Gynecol. (2008) 111:1446. 10.1097/AOG.0b013e31817aff21 18515534

[3] Miller DA Chollet JA Goodwin TM. Clinical risk factors for placenta previa–placenta accreta. Am J Obstet Gynecol. (1997) 177:210–4. 10.1016/S0002-9378(97)70463-0 9240608

[4] Matsuzaki S Mandelbaum RS Sangara RN McCarthy LE Vestal NL Klar M Trends, characteristics, and outcomes of placenta accreta spectrum: a national study in the United States. Am J Obstet Gynecol. (2021)225(5):534. e1–534 e38. 10.1016/j.ajog.2021.04.233 33894149

[5] Clark SL Koonings PP Phelan JP. Placenta previa/accreta and prior cesarean section. Obstet Gynecol. (1985) 66:89–92. 4011075

[6] Placenta Accreta Spectrum | ACOG. https://www.acog.org/clinical/clinical-guidance/obstetric-care-consensus/articles/2018/12/placenta-accreta-spectrum (Accessed February 18, 2021) (n.d.).

[7] Seet EL Kay HH Wu S Terplan M. Placenta accreta: depth of invasion and neonatal outcomes. J Matern Neonatal Med. (2012) 25:2042–5. 10.3109/14767058.2012.678429 22463851

[8] Warshak CR Ramos GA Eskander R Benirschke K Saenz CC Kelly TF Effect of predelivery diagnosis in 99 consecutive cases of placenta accreta. Obstet Gynecol. (2010) 115:65–9. 10.1097/AOG.0b013e3181c4f12a 20027036

[9] Chantraine F Braun T Gonser M Henrich W Tutschek B. Prenatal diagnosis of abnormally invasive placenta reduces maternal peripartum hemorrhage and morbidity. Acta Obstet Gynecol Scand. (2013) 92:439–44. 10.1111/aogs.12081 23331024

[10] Tsirulnikov MS. Ligation of the uterine vessels during obstetrical hemorrhages. Immediate and long-term results (author's Transl). J Gynecol Obstet Biol Reprod (Paris). (1979) 8:751–3. 317962

[11] Cho JH Jun HS Lee CN. Hemostatic suturing technique for uterine bleeding during cesarean delivery. Obstet Gynecol. (2000) 96:129–31. 10.1016/S0029-7844(00)00852-8 10928901

[12] Bretelle F Courbière B Mazouni C Agostini A Cravello L Boubli L Management of placenta accreta: morbidity and outcome. Eur J Obstet Gynecol Reprod Biol. (2007) 133:34–9. 10.1016/j.ejogrb.2006.07.050. 16965851

[13] Kerber C. Balloon catheter with a calibrated leak. Radiology. (1976) 120:547–50. 10.1148/120.3.547 948586

[14] Kelly HA. Ligation of both internal iliac arteries for hemorrhage in hysterectomy for carcinoma uteri. Journal de gynécologie, obstétrique et biologie de la reproduction. (1984) 20:248.

[15] Abbas AM Shady NW Sallam HF. Bilateral uterine artery ligation plus intravenous tranexamic acid during cesarean delivery for placenta previa: a randomized double-blind controlled trial. J Gynecol Obstet Hum Reprod. (2019) 48:115–9. 10.1016/j.jogoh.2018.10.023 30412786

[16] El Gelany S Ibrahim EM Mohammed M Abdelraheim AR Khalifa EM Abdelhakium AK Management of bleeding from morbidly adherent placenta during elective repeat caesarean section: retrospective -record -based study. BMC Pregnancy Childbirth. (2019) 19:1–7. 10.1186/s12884-019-2244-4 30922265 PMC6439998

[17] Book Series C, Higgins JP Green S. Cochrane handbook for systematic reviews of interventions the cochrane collaboration®. (n.d.) United kingdom, cochrane collaboration: Wiley online library.

[18] Moher D Liberati A Tetzlaff J Altman DG. Preferred reporting items for systematic reviews and meta-analyses: the PRISMA statement. Br Med J. (2009) 339:332–6. 10.1136/bmj.b2535 PMC3090117 21603045

[19] Page MJ McKenzie JE Bossuyt PM Boutron I Hoffmann TC Mulrow CD The PRISMA 2020 statement: an updated guideline for reporting systematic reviews. Br Med J. (2021):372:n71. 10.1136/BMJ.N71 PMC8005924 33782057

[20] Stroup DF Berlin JA Morton SC Olkin I Williamson GD Rennie D Meta-analysis of observational studies in epidemiology: a proposal for reporting. Meta-analysis of observational studies in epidemiology (MOOSE) group. JAMA. (2000) 283:2008–12. 10.1001/JAMA.283.15.2008 10789670

[21] Study Quality Assessment Tools | NHLBI, NIH n.d.

[22] Hussein AM Dakhly DMR Raslan AN Kamel A Abdel Hafeez A Moussa M The role of prophylactic internal iliac artery ligation in abnormally invasive placenta undergoing caesarean hysterectomy: a randomized control trial. J Matern Neonatal Med. (2019) 32:3386–92. 10.1080/14767058.2018.1463986 29635951

[23] Shmakov RG Vinitskiy AA Chuprinin VD Yarotskaya EL Sukhikh GT. Alternative approaches to surgical hemostasis in patients with morbidly adherent placenta undergoing fertility-sparing surgery. J Matern Neonatal Med. (2019) 32:2042–8. 10.1080/14767058.2018.1424821 29402157

[24] Köstü B Özer A Ercan Ö Bakacak M Karakuş S Keten H. Prophylactic hypogastric artery ligation in surgery for placental invasion disorders. Int J Clin Exp Med. (2016) 9:16735–40. 10.1055/s-0038-1666793

[25] Lin J Lin F Zhang Y. Uterine artery ligation before placental delivery during caesarean in patients with placenta previa accreta. Med (United States). (2019) 98:1–4. 10.1097/MD.0000000000016780 PMC6739010 31490365

[26] El Gelany S Mosbeh MH Ibrahim EM Mohammed M Khalifa EM Abdelhakium AK Placenta accreta Spectrum (PAS) disorders: incidence, risk factors and outcomes of different management strategies in a tertiary referral hospital in minia, Egypt: a prospective study. BMC Pregnancy Childbirth. (2019) 19:313. 10.1186/s12884-019-2466-5 31455286 PMC6712589

[27] Kuhn T Martimucci K Al-Khan A Bilinski R Zamudio S Alvarez-Perez J. Prophylactic hypogastric artery ligation during placenta percreta surgery: a retrospective cohort study. Am J Perinatol Rep. (2018) 08:e142–5. 10.1055/s-0038-1666793 PMC6023712 29977660

[28] Sucu S Özcan HÇ Karuserci ÖK Demiroğlu Ç Tepe NB Bademkıran MH. Is there a role of prophylactic bilateral internal iliac artery ligation on reducing the bleeding during cesarean hysterectomy in patients with placenta percreta? A Retrospective Cohort Stud. (2020) 91:1–6. 10.5603/GP.a2020.0145 33448009

[29] Barinov S Tirskaya Y Medyannikova I Shamina I Shavkun I. A new approach to fertility-preserving surgery in patients with placenta accreta. J Matern Neonatal Med. (2019) 32:1449–53. 10.1080/14767058.2017.1408066 29157035

[30] Iwata A. Limitations of internal iliac artery ligation for the reduction of intraoperative hemorrhage during cesarean hysterectomy in cases of placenta previa accreta. Obstet Gynaecol Res. (2010) 36:254–9. 10.1111/j.1447-0756.2009.01157.x 20492374

[31] Ono Y Murayama Y Era S Matsunaga S Nagai T Osada H Study of the utility and problems of common iliac artery balloon occlusion for placenta previa with accreta. J Obstet Gynaecol Res. (2018) 44:456–62. 10.1111/jog.13550 29297951 PMC5873444

[32] Joshi VM Otiv SR Majumder R Nikam YA Shrivastava M. Internal iliac artery ligation for arresting postpartum haemorrhage. BJOG An Int J Obstet Gynaecol. (2007) 114:356–61. 10.1111/j.1471-0528.2006.01235.x 17261120

[33] İçen MS Findik FM Akin Evsen G Ağaçayak E Yaman Tunç S Evsen MS Hypogastric artery ligation in postpartum haemorrhage: a ten-year experience at a tertiary care centre. J Obstet Gynaecol. (2021) 41:536–40. 10.1080/01443615.2020.1755623 32496842

[34] Bakacak Z Bakacak M Uzkar A Yazar FM Yaylalı A Boran ÖF The efficacy of LigaSure™ open instruments in cases of cesarean hysterectomy due to placenta percreta: a retrospective, record-based, comparative study. J Matern Neonatal Med. (2021) 34:960–5. 10.1080/14767058.2020.1846177 33256477

[35] Bakri YN. Uterine tamponade-drain for hemorrhage secondary to placenta previa-accreta. Int J Gynecol Obstet. (1992) 37:302–3. 10.1016/0020-7292(92)90336-H 1350550

[36] Papillon-Smith J Hobson S Allen L Kingdom J Windrim R Murji A. Prophylactic internal iliac artery ligation versus balloon occlusion for placenta accreta spectrum disorders: a retrospective cohort study. Int J Gynecol Obstet. (2020) 151:91–6. 10.1002/ijgo.13256 32506473

[37] Wei Y Luo J Luo D. Comparison of efficacy between internal iliac artery and abdominal aorta balloon occlusions in pernicious placenta previa patients with placenta accrete. Gynecol Obstet Invest. (2019) 84:343–9. 10.1159/000494493 30625467

