# Supplementary material for: Therapeutic effect of Internal iliac artery ligation and uterine artery ligation techniques for bleeding control in placenta accreta spectrum patients: A meta-analysis of 795 patients
Source: Front Surg. 2022 Sep 1;9:983297. doi: 10.3389/fsurg.2022.983297 (PMC9474733; doi:10.3389/fsurg.2022.983297)
Supplement: Supplementary file 1 [file Table_1_v1.docx]

Cohort studies and articles that didn't specify exactly blood loss assessment methods

2.1 blood loss
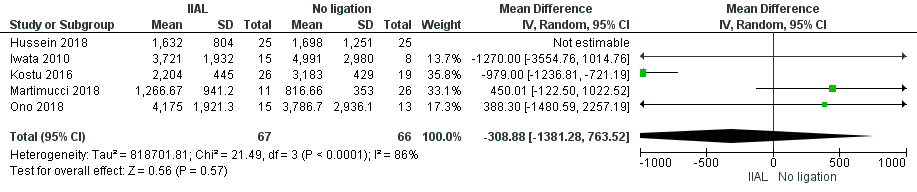
2.2 change from baseline in HgB
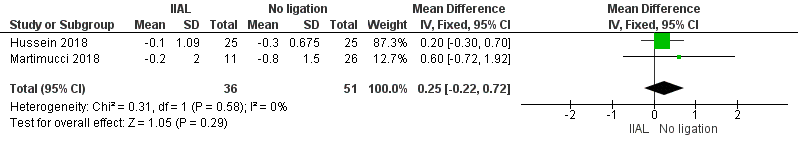
2.3 blood transfusion


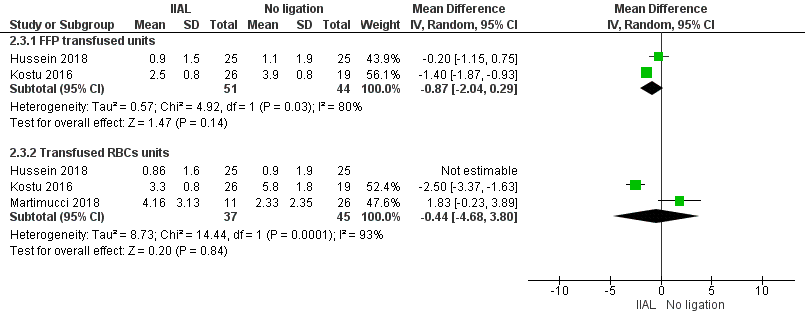


2.4 complications
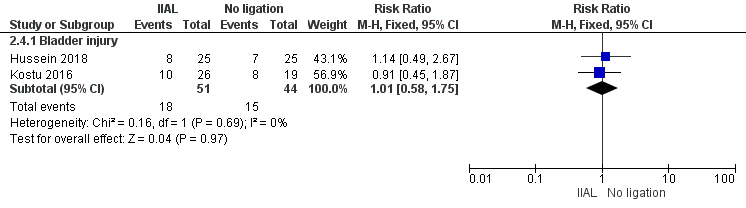
3.1 blood loss
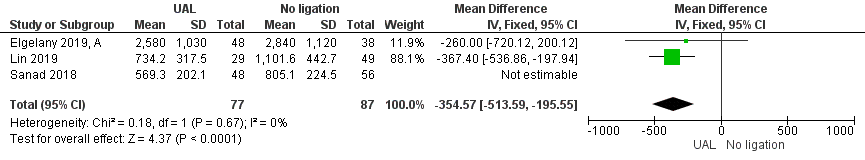
3.2 change from baseline in HgB
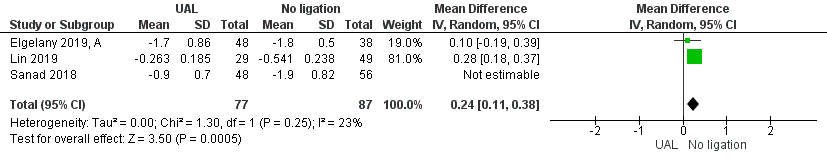
3.3 complications
